# Effect of virtual reality distraction on pain and anxiety during infiltration anesthesia in pediatric patients: a randomized clinical trial

**DOI:** 10.1186/s12903-021-01678-x

**Published:** 2021-06-25

**Authors:** Osama M. Felemban, Rawan M. Alshamrani, Doha H. Aljeddawi, Sara M. Bagher

**Affiliations:** 1 Pediatric Dentistry Department, Faculty of Dentistry, King Abdulaziz University, Jeddah, Saudi Arabia; 2 Ministry of Health, Riyadh, Saudi Arabia; 3 Ministry of Health, Madinah, Saudi Arabia

**Keywords:** Anxiety, Distraction, Infiltration anesthesia, Pain, Virtual reality

## Abstract

**Background:**

Different distraction techniques have been used in dentistry and have shown great results in managing anxious pediatric patients specially during local anesthesia administration. One of the recently invented techniques is virtual reality. The purpose of the study was to evaluate the effect of virtual reality distraction on anxiety and pain during buccal infiltration anesthesia in pediatric patients.

**Methods:**

Healthy, cooperative 6- to 12-year-old children requiring buccal infiltration anesthesia were randomly assigned to a test or control group. In the test group, local anesthesia was administered while the subjects were watching a cartoon video using virtual reality goggles. Subjects in the control group watched a cartoon video on a screen during the administration of local anesthesia. To assess anxiety in both groups, heart rate was recorded using a pulse oximeter at five time points: (1) once the subject sets on the dental chair as a baseline; (2) when video is on; (3) at topical anesthesia application; (4) during needle insertion; (5) after the administration of local anesthesia. The face, legs, activity, cry, consolability (FLACC) behavioral pain assessment scale and the Wong–Baker FACES pain rating scale were used to assess pain.

**Results:**

A total of 50 subjects were included with a mean age of 8.4 ± 1.46 years. Twenty-nine (58.0%) of the subjects were females. The mean heart rate at all time points except baseline was significantly higher among the test group compared to the control group. Multiple regression analysis showed that younger subjects and females had higher mean FLACC behavioral pain assessment scale scores (*P* = 0.034 and *P* = 0.004, respectively) regardless of the distraction technique used. Younger subjects and subjects with higher baseline heart rate reported higher mean Wong–Baker FACES pain rating scale score (*P* = 0.031 and *P* = 0.010, respectively), controlling for all other variables.

**Conclusion:**

Female subjects and the younger age group were more likely to report higher pain scores during local anesthesia administration regardless of the type of distraction used.

***Trial registration*:**

The study was retrospectively registered in ClinicalTrials.gov with the identifier: NCT04483336 on 23/07/2020.

## Background

Proper pain control and discomfort reduction during dental treatment, especially among children, can maximize a child’s cooperation, overall satisfaction, build a good dentist–patient relationship, and enhance patient compliance [1]. It has been reported that the local anesthetic injection is the most fearful part of a dental visit. Local anesthesia is associated with high levels Fof anxiety which highlights the important role of behavior management when treating children [2]. Conventional modalities of behavior guidance of pediatric dental patients include simple methods such as tell, show, and do; positive reinforcement; voice control; and distraction. Behavior guidance modalities also include more advanced techniques such as protective stabilization, treatment under conscious sedation, or general anesthesia [1].

Distraction as a behavior guidance technique is defined by the American Academy of Pediatric Dentistry (AAPD) as “the technique of diverting the patient’s attention from what may be perceived as an unpleasant procedure” [1]. Audiovisual distraction techniques are used in dental clinics and have shown great results in managing anxious pediatric patients [3, 4]. Virtual reality (VR) distraction, defined as “a human–computer interface that enables the user to interact dynamically with the computer-generated environment” is a new method in the medical field with the aim of aiding in patient behavior management. It offers the advantage of an immersive virtual experience blocking out external stimuli that may provoke a negative attitude, especially in young patients [5]. Distraction using VR provided favorable outcomes for adult and pediatric patients during various dental procedures, ranging from simple anesthesia to periodontal, restorative, and pulpal therapy [6–12].

A published study testing the effect of VR distraction during inferior alveolar nerve block and pulpal therapy showed significantly more reduction in pain among subjects who received VR distraction when compared with subjects who were treated without the use of VR distraction [13]. Although pediatric patients behave more positively toward inferior alveolar nerve block compared with buccal infiltration injection, no studies were specific to buccal infiltration anesthesia [14]. Our study aimed to evaluate the effect of virtual reality distraction on anxiety and pain during buccal infiltration anesthesia in pediatric patients. We hypothesized that virtual reality distraction will decrease anxiety and pain during buccal infiltration anesthesia in pediatric patients.

## Methods

This parallel randomized controlled trial was conducted in the Pediatric Dentistry department of King Abdulaziz University, Faculty of Dentistry (KAUFD) between March and July 2019. The study protocol was approved by the Research Ethics Committee of the Faculty of dentistry (REC-FD) at King Abdulaziz University (136-11-18). The study was registered in ClinicalTrials.gov with the identifier: NCT04483336. Reporting of the study follows the protocol established by the Consolidated Standards of Reporting Trials Statement (CONSORT) checklist [15]. Healthy and cooperative 6- to 12-year-old children, with no known allergy and/or sensitivity to local anesthesia who needed non-urgent dental treatment under local anesthesia buccal infiltration by one of the postgraduate or interns at the pediatric dentistry students, were eligible for the study. Patients with a history of epilepsy and anxiety disorder were excluded from the study. Parents/guardians of eligible subjects who agreed to be enrolled in the study signed an informed consent document in Arabic language before participation. Subjects’ age, gender, previous dental experience, and behavior during previous dental treatment based on Frankl’s behavior rating scale classification were recorded in addition to whether or not they own a VR device.

A randomization sequence with an allocation ratio of 1:1 was generated using computer software and was kept with a dental assistant who was not involved in the data collection to ensure allocation concealment. Each time a new subject was enrolled in the study, the dental assistant was asked to provide the allocation assignment of the subject. Due to the nature of the study, neither the subject nor the investigators were blinded to the group allocation. However, the group allocation was coded by the primary investigator to blind the statistician of the group labels.

**Fig. 1:**
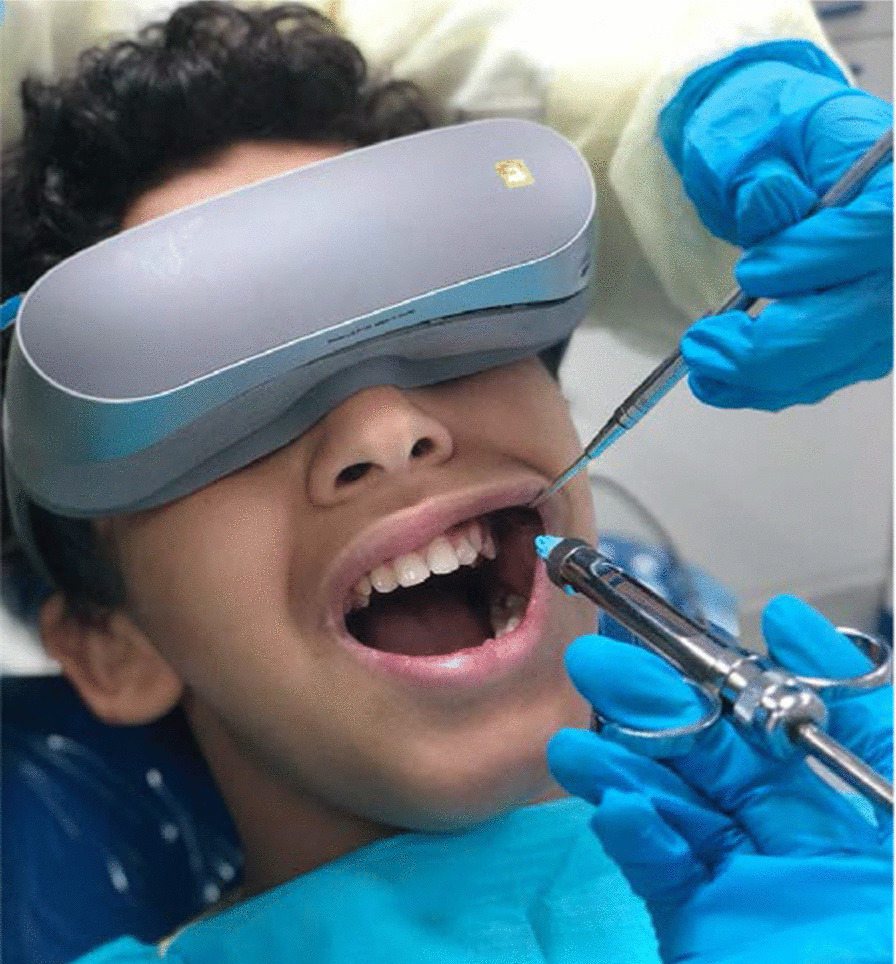
Use of virtual reality goggles for distraction during buccal infiltration anesthesia

At the beginning of the dental visit, subjects were asked to choose a video from a list of popular cartoon shows. In the test group, the subjects wore VR goggles (LG 360 virtual reality [VR] headset, LG Electronics) connected to a mobile phone and the chosen video was played (Fig. 1). In the control group, the chosen video was played on a screen connected to a computer attached to the dental chair. The sounds of the videos were played on the speakers of the mobile phone (test group) or the computer (control group). No headphones were used in either group. A buccal infiltration local anesthesia was administered to all subjects as follows: the mucosa was dried with cotton rolls; 20% benzocaine topical anesthetic gel (Sky-Caine Gel, Skydent Inc., NY, USA) was applied for 20 s, followed by injection of mepivacaine 2% with 1/100,000 epinephrine (Scandicaine 2% speciale, Septodont, UK) using 27G short needle (Septoject XL, Septodont, UK). The anesthesia was provided by the dental provider of the subject (either postgraduate pediatrics dental students or dental interns).

Subjects’ heart rates (HR) were recorded using a pulse oximeter (OxyWatch, ChoiceMMed, Hamburg, Germany) at five time points: (1) once the subject is on the dental chair as a baseline; (2) when video is on (about 3 min later); (3) at topical anesthesia application (about 2 min later); (4) at needle insertion (about 2 min later); (5) immediately after the administration of local anesthesia (about 1 min later).

**Fig. 2:**
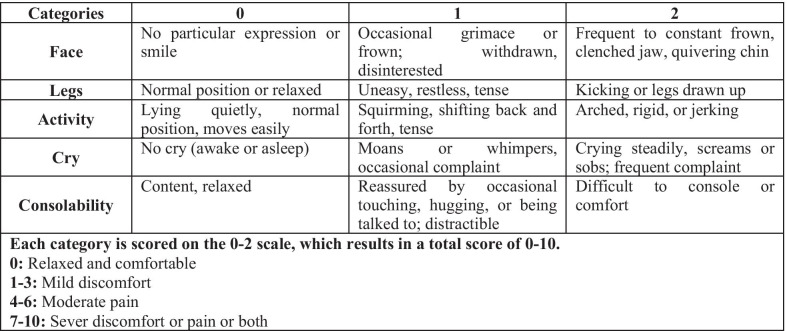
The face, legs, activity, cry, consolability (FLACC) behavioral pain assessment scale

During local anesthesia administration, the face, legs, activity, cry, consolability (FLACC) behavioral pain assessment scale [16] was recorded by two trained and calibrated investigators independently to assess pain (Fig. 2). Immediately after anesthesia administration, the subjects were placed in an upright position and were shown the Arabic version of Wong–Baker FACES pain rating scale and they were asked to pick the face that described their experienced feeling during the administration of the local anesthesia [17].

Sample size was calculated using the data of HR from Nuvvula et al. [9] It was found that 25 subjects per group were required to have a statistically significant difference between the two groups at 0.05 significance level and 95% power. Based on the studies of Bagattoni et al. [8], Aminabadi et al. [7], Shetty et al. [10], and Panda [13], it was estimated that the mean score for the Wong–Baker FACES pain rating scale to be about 2.07 ± 1.55 in the test group and 3.97 ± 0.93 in the control group. It was estimated that 12 subjects in each group were needed to detect a statistically significant difference at the level of 0.05 with a power of 95%. No data were found to use the FLACC behavioral pain assessment scale in sample size calculation. Since the two calculations yielded different required sample sizes, the larger number per group (*n* = 25) was set as the required sample size. GPower 3.1.9.2 software was used for sample size calculation. The Mann–Whitney U and Chi-square tests were used to compare the baseline characteristics, HR, FLACC behavioral pain assessment scale, and the Wong–Baker FACES pain rating scale score between the test and the control groups at 0.05 significance level. Since HR was measured repeatedly, a two-way mixed ANCOVA test was used to check for interaction effects among age, gender, group, and time points with HR as the dependent variable. Multiple linear regression analysis was used to predict the effects of VR on HR and FLACC behavioral pain assessment scale controlling for possible confounding by age, gender, and the baseline HR. Similarly, ordinal logistic regression was used to model the effects of VR on Wong–Baker FACES pain rating scale scores, to check for interactions, and to control for possible confounding by age, gender, and the baseline HR.

## Results

**Fig. 3:**
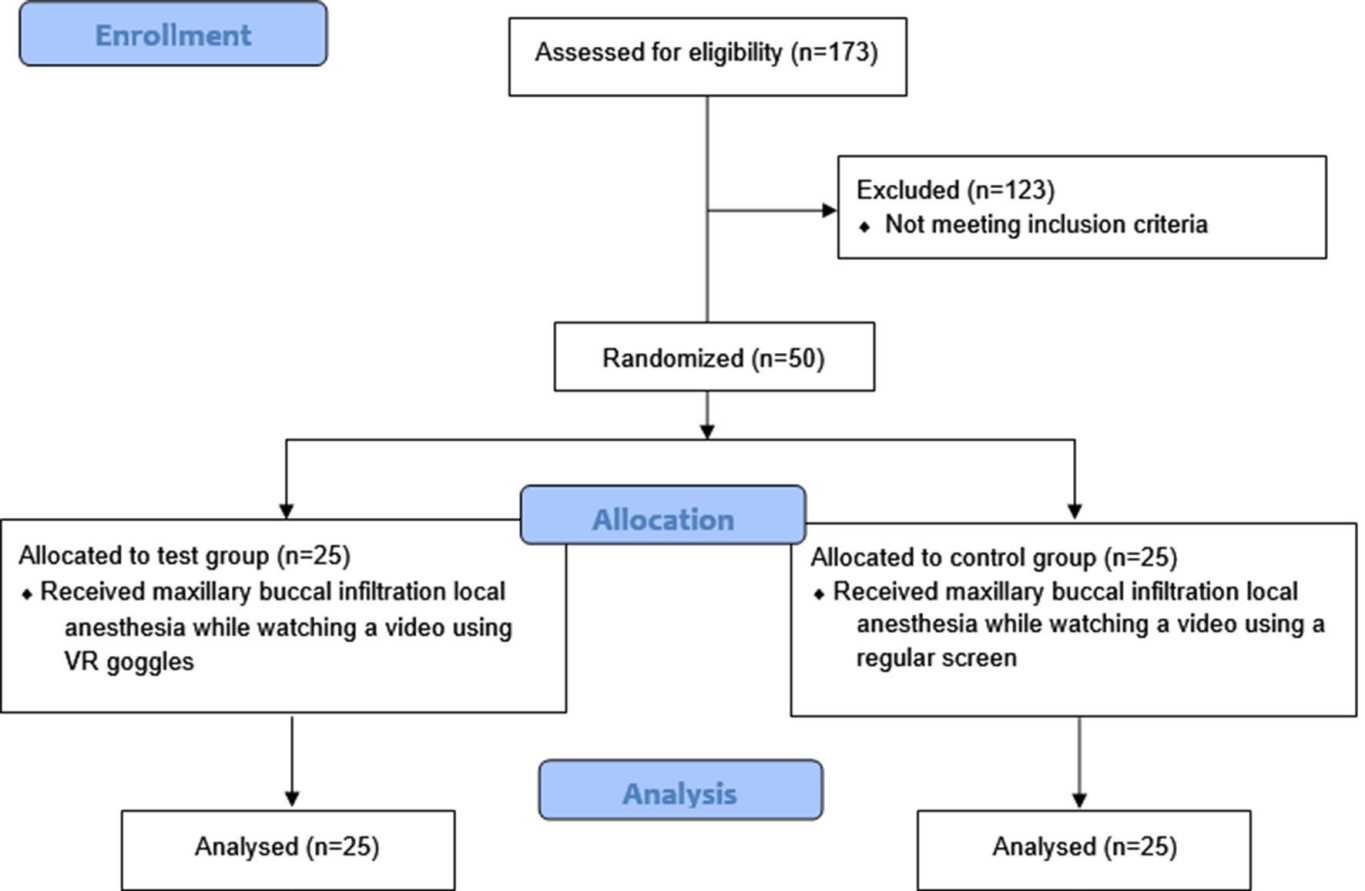
CONSORT flow diagram of recruitment of subjects, randomization, allocation, completion of local anesthesia administration, and analysis

An illustration of the subjects’ recruitment, randomization, allocation, completion of local anesthesia administration, and analysis are represented in the CONSORT flow diagram (Fig. 3). A total of 50 subjects (25 in the test group and 25 in the control group) participated in the study (Table 1). Twenty-two (44.0%) of the subjects were between the ages of 6–8 years and 28 subjects (65.0%) were between the ages of 9–12 years. More than half the subjects were males (58.0%), while females accounted for 42.0%. Eight percent of the subjects also did not have previous dental experience. Of those who had previous dental experience, more than half were classified as definitely positive (60.0%) and only 18 subjects (39.1%) were classified as positive based on Frankl’s behavior rating scale classification in previous dental visits. More than two thirds (68.0%) of the local anesthesia procedures were given in the maxillary arch. About half of the procedures were performed by postgraduate students (48.0%) while dental interns performed 52.0% of the procedures. The majority of subjects did not own a VR device (98.0%). No significant differences at baseline were found between the test and control group in terms of age (*P* = 0.254), gender (*P* = 0.774), provider (postgrad vs. intern) (*P* = 0.258), location of local anesthesia (maxilla versus mandible) (*P* = 0.544), owning a VR device (*P* = 1.00), previous dental experience (*P* = 1.00), or Frankl’s behavior classification for those who had previous dental experience (*P* = 1.00).

**Table 1:** Demographic characteristics of the participants (*n* = 50)

Variables	Total	Test	Control	*P*-value‡
	*n* (%)	*n* (%)	*n* (%)	
*Age*				
Younger (6–8 years)	22 (44.0)	13 (52.0)	9 (36.0)	0.254
Older (9–12 years)	28 (56.0)	12 (48.0)	16 (64.0)	
*Gender*				
Male	29 (58.0)	14 (56.0)	15 (60.0)	0.744
Female	21 (42.0)	11 (44.0)	10 (40.0)	
*Previous dental experience*				
Yes	46 (92.0)	23 (92.0)	23 (92.0)	1.00
No	4 (8.0)	2 (8.0)	2 (8.0)	
*Frankl behavior rating scale of those who had previous dental experience (n* = *46)*				
Definitely positive	28 (60.9)	14 (60.9)	14 (60.9)	1.00
Positive	18 (39.1)	9 (39.1)	9 (39.1)	
*Location of procedure*				
Maxilla	34 (68.0)	16 (64.0)	18 (72.0)	0.544
Mandible	16 (32.0)	9 (36.0)	7 (28.0)	
*Provider*				
Postgrad	24 (48.0)	10 (40.0)	14 (56.0)	0.258
Intern	26 (52.0)	15 (60.0)	11 (44.0)	
*Own a VR device*				
Yes	1 (2.0)	1 (4.0)	0 (0.0)	1.00
No	49 (98.0)	24 (96.0)	25 (100%)	

*VR* virtual reality

^‡^Comparison of test and control groups using Chi-square test or Fisher exact test if one of the cells had a frequency of less than 5

Table 2 depicts the pain and anxiety scores for the study participants. The mean HR at baseline was higher in the test group (91.20 ± 14.53) compared with the control group (85.48 ± 9.98), but the difference was not statistically significant (*P* = 0.153). Although there was a statistically significant difference between the test and control groups in the mean HR at video on (*P* = 0.012), at topical anesthesia application (*P* = 0.047), at needle insertion (*P* = 0.017), and after buccal infiltration anesthesia (*P* = 0.001), the mean change in HR from the baseline to these time points was not statistically different between the test and control groups (*P* = 0.228, *P* = 0.984, *P* = 0.437, and *P* = 0.111, respectively). The mean score of the FLACC behavioral pain assessment scale measured after the local anesthesia procedure was slightly higher among the test group (2.58 ± 1.99) compared with the control group (2.18 ± 2.29); however, the difference was not statistically significant (*P* = 0.497). The mean Wong–Baker FACES pain rating scale score was lower among the test group (2.40 ± 2.82) compared with the control group (2.72 ± 2.99); however, the difference was not statistically significant (*P* = 0.707).

**Table 2:** Anxiety and pain mean scores of the participants (*n* = 50)

Variables	Test	Control	*p*-value†
	Mean ± SD	Mean ± SD	
*Heart rate per minute*			
Baseline	91.20 ± 14.53	85.48 ± 9.98	0.153
Video on	94.20 ± 14.62	84.00 ± 10.62	0.012*
Topical anesthesia application	93.20 ± 13.44	86.36 ± 12.29	0.047*
Needle insertion	100.00 ± 15.52	89.44 ± 13.59	0.017*
Immediately after local anesthesia	104.08 ± 15.34	90.20 ± 14.29	0.001*
HR difference (video on—baseline)	3.00 ± 12.45	− 1.48 ± 8.00	0.228
HR difference (topical anesthesia—baseline)	2.00 ± 12.52	0.88 ± 11.62	0.984
HR difference (needle insertion—baseline)	8.80 ± 17.38	3.96 ± 11.61	0.437
HR difference (after local anesthesia—baseline)	12.88 ± 16.74	4.72 ± 14.41	0.111
FLACC behavioral pain assessment scale	2.58 ± 1.99	2.18 ± 2.29	0.497
Wong–Baker FACES pain rating scale	2.40 ± 2.82	2.72 ± 2.99	0.707

^†^Mann–Whitney U test

^*^Statistically significant

Since HR was measured at five different time points in each group, two-way mixed ANCOVA was used to test if interactions existed among the variables of time, age, gender, and group. The results showed no statistically significant interactions between time*group (*P* = 0.347), time*gender (*P* = 0.223), or time*age (*P* = 0.592). The effect of using VR on changes in the HR from the baseline to each time point was further analyzed using multiple linear regression to control for possible confounding by age, gender, and baseline HR and is presented in Table 3. In general, higher baseline HR was associated with a significant decrease in HR difference at all time points controlling for age, gender, and group. Subjects in the test group had significantly higher change in the HR at video on by 6.49 beats per minute (0.60–12.38) compared with subjects in the control group controlling for age, gender, and HR at the baseline. Additionally, subjects in the test group had statistically significant higher change in HR from the baseline to after local anesthesia administration by 10.68 beats per minute (2.58–18.79) compared with the control group.

**Table 3:** Multiple linear regression analysis for the prediction of heart rate changes (adjusted model)

Variable	Category	HR difference (video on—baseline) R^2^ = 0.195	HR difference (topical anesthesia application— baseline) R^2^ = 0.267	HR difference (needle insertion—baseline) R^2^ = 0.232	HR difference (after local anesthesia—baseline) R^2^ = 0.331
		β ± SE (95% CI) *P*-value	β ± SE (95% CI) *P*-value	β ± SE (95% CI) *P*-value	β ± SE (95% CI) *P*-value
Age	6–8 years	− 0.64 ± 2.90 (− 6.48 to 5.20) 0.827	2.26 ± 2.89 (− 3.56 to 8.08) 0.438	2.91 ± 3.96 (− 5.07 to 10.88) 0.467	3.46 ± 3.99 (− 4.57 to 11.50 0.390
	9–12 years	Reference	Reference	Reference	Reference
Gender	Females	− 1.12 ± 2.88 (− 6.92 to 4.68) 0.700	− 0.79 ± 2.87 (− 6.57 to 4.99) 0.785	2.59 ± 3.93 (− 5.33 to 10.52) 0.513	7.01 ± 3.96 (− 0.97 to 14.99) 0.084
	Males	Reference	Reference	Reference	Reference
HR baseline		− 0.33 ± 0.12 (− 0.56 to − 0.09) 0.007*	− 0.44 ± 0.12 (− 0.67 to − 0.21) < 0.001*	− 0.52 ± 0.16 (− 0.84 to − 0.20) 0.002*	− 0.59 ± 0.16 (− 0.91 to − 0.27) 0.001*
Group	Test	6.49 ± 2.93 (0.60 to 12.38) 0.032*	4.65 ± 2.92 (− 1.23 to 10.52) 0.118	7.24 ± 3.99 (− 0.81 to 15.28) 0.077	10.68 ± 4.03 (2.58 to 18.79) 0.011*
	Control	Reference	Reference	Reference	Reference

*HR* heart rate

^*^Statistically significant

Multiple linear regression analysis was also used to evaluate the effect of VR on the mean FLACC behavioral pain assessment scale of subjects controlling for age, gender, and the baseline HR. All two-way interactions were checked, and no significant interaction between the predictor variables was found. In the unadjusted model, younger children compared with older children and females compared with males had significantly higher mean scores on the FLACC behavioral pain assessment scale (*P* = 0.017 and *P* = 0.001, respectively). In the adjusted model, younger subjects had a statistically significant higher mean score on the FLACC behavioral pain assessment scale by 1.20 (0.09–2.30) compared with older subjects controlling for gender, the baseline HR, and group. Also, females had a significantly higher mean score on the FLACC behavioral pain assessment scale by 1.68 (0.58–2.78) compared with males controlling for all other variables. Being in the test group did not have a statistically significant effect on the mean FLACC behavioral pain assessment scale when other predictors were controlled for (Table 4).

**Table 4:** Linear regression model for the prediction of FLACC behavioral pain assessment scale (unadjusted and adjusted)

Variable	Category	Unadjusted model				Adjusted model (R^2^ = 0.290)		
		β ± SE	(95% CI)	*P*-value	R^2^	β ± SE	(95% CI)	*P*-value
Age	6–8 years	1.43 ± 0.58	(0.27 to 2.60)	0.017*	0.113	1.20 ± 0.55	(0.09 to 2.30)	0.034*
	9–12 years	Reference				Reference		
Gender	Females	1.89 ± 0.56	(0.78 to 3.01)	0.001*	0.195	1.68 ± 0.55	(0.58 to 2.78)	0.004*
	Males	Reference				Reference		
HR baseline		0.03 ± 0.02	(− 0.02 to 0.08)	0.266	0.026	0.02 ± 0.02	(− 0.02 to 0.07)	0.293
Group	Test	0.40 ± 0.61	(− 0.82 to 1.62)	0.513	0.009	0.01 ± 0.55	(− 1.11 to 1.12)	0.988
	Control	Reference				Reference		

*HR* heart rate

^*^Statistically significant

Regarding the results of the Wong–Baker FACES pain rating scale, an ordinal logistic regression was used to model the effects of VR on the pain scale controlling for age, gender, and baseline HR. None of the two-way interactions was found to be significant. In the unadjusted model, only the baseline HR was found to be associated with higher scores of the Wong–Baker FACES pain rating scale (OR 1.05; 95% CI 1.00–1.09; *P* = 0.039). The baseline HR was also associated with higher scores of the pain rating scale even when controlling for age, gender, and group (*P* = 0.010). In addition, younger subjects were significantly more likely to report higher pain scores of the Wong–Baker FACES pain rating scale (OR 3.37; 1.12–10.19; *P* = 0.031) compared with older subjects controlling for other variables in the model. The use of VR did not have significant effect on pain scores (*P* = 0.188) adjusting for age, gender, and the baseline HR (Table 5).

**Table 5:** Ordinal logistic regression for the prediction of Wong–Baker pain rating scale scores (unadjusted and adjusted)

Variable	Category	Unadjusted model			Adjusted model		
		OR	(95% CI)	*P*-value	OR	(95% CI)	*P*-value
Age	6–8 years	2.22	(0.79–6.29)	0.132	3.37	(1.12–10.19)	0.031*
	9–12 years	Reference			Reference		
Gender	Females	0.74	(0.26–2.08)	0.571	0.55	(0.19–1.62)	0.278
	Males	Reference			Reference		
HR baseline		1.05	(1.00−1.09)	0.039*	1.07	(1.02−1.12)	0.010*
Group	Test	0.82	(0.30–2.25)	0.704	0.48	(0.16–1.44)	0.188
	Control	Reference			Reference		

*HR* heart rate

^*^Statistically significant

## Discussion

Unpleasant previous dental experience, including the administration of local anesthesia, was found to be associated with higher levels of dental fear, uncooperative behaviors during the treatment [18] and the possibility of avoidance of dental visits in the future [19]. Thus, it is imperative for dental practitioners to establish a pleasant dental environment for their patients in order to deliver comprehensive and continuous optimal oral health care. The aim of our study was to evaluate the effect of VR distraction on anxiety and pain in children during the administration of infiltration anesthesia. Our study targeted the age group of 6- to 12-year-old children because this is the age group that can appreciate the joy and excitement of putting on a VR device and are capable of being immersed in the experience. They are also old enough to answer questions about pain levels independently. We excluded children with epilepsy, as empirical evidence suggested that virtual reality devices may trigger seizure episodes in photosensitivity-susceptible individuals [20]. To differentiate between the effects of video distraction and VR distraction, the controls were shown a cartoon video on a regular screen.

Three methods were chosen to measure anxiety and pain: HR as a physiological measurement of anxiety; FLACC behavioral pain assessment scale as an objective measurement; and Wong Baker FACES pain rating scale as a subjective measurement for pain. The HR was used to express the level of anxiety during different steps of anesthesia administration. Heart rate is considered a valid and sensitive measure for assessment of anxiety in children during various dental procedures; further, the use of heart rate to measure and detect changes in the degree of anxiety in children during dental treatment, especially in research of children’s dental behavior, is increasing [21].

The results of our study showed that, although the subjects were randomly allocated, the baseline HR in the test group was higher than in the control group, which occurred most probably due to chance. Therefore, it was important to control for this difference in the subsequent analysis.

Also, once the VR device was turned on, there was significantly more increase in the HR among subjects in the test group when compared with the HR of the subjects in the control group when the screen was turned on, which agrees with a previous study in which an elevation of HR among users of the VR device was reported [22]. Although the mean levels of HR were significantly higher in the test group at different points during the local anesthesia procedure compared with the control group, the amount of change in HR from the baseline to the different time points was not statistically significantly different between the two groups. The regression analysis of HR change indicated that higher HR at baseline decreased the change in HR, while the VR increased the change in HR when age and gender were controlled for. This indicates that VR caused a significant increase in HR during local anesthesia administration in comparison with the screen distraction used in the control group when controlling for age and gender. These results are in agreement with two previous studies, which compared audiovisual eyeglasses with the use of tablets as distraction devices and reported that the tablet distraction was associated with a higher increase in the HR [6, 23]. Other studies showed that HR decreased with the use of VR distraction, but their control group received treatment with no distraction [24, 25]. Three recent meta-analyses showed conflicting results. Zhang et al. [26] results showed that HR decreases when audiovisual distraction is used while Liu et al. [27] showed that audiovisual distraction did not significantly change heart rate. Custodio et al., in comparing virtual reality glasses to other distraction techniques during local anesthesia administration, revealed that there was no significant difference in heart rates [28].

The increased level of HR among the test group could be because the study evaluated pain during aesthesia application which is considered one of the main fear-provoking procedures among children [19]. Also, most of our study subjects had no previous exposure to VR eyeglasses, making it a new experience that may result in excitement. Another reason is that most subjects had previous dental experience and are aware that they will most probably receive anesthesia. Having their vision blocked by the VR goggles may make them feel isolated from the real word and increase their level of anticipation of an unpleasant stimulus and, consequently, their HR. Moreover, it was shown that distraction methods are less effective in patients with previous pain experience [29].

The application of VR systems can be challenging during dental treatment especially if the size and position of the VR goggles could obstruct the dentist field of work. The VR goggles chosen in our study was small in size and light weighted. Recent advances in VR technology could provide a more powerful immersive experience and potentially a higher distraction from pain and anxiety. In a recent pilot laboratory study, eye tracking technology was embedded into the VR system allowing the participants to interact with the virtual world using their eye movements. This technology significantly increased the participants illusion and was more effective in increasing the analgesic effectiveness when compared to the regular VR systems [30]. Applying such VR system during dental treatment can create a stronger illusion and therefore be more effective in reducing pain.

Two measures were used for the assessment of pain: the FLACC behavioral pain assessment scale and Wong–Baker FACES pain rating scale. Many studies support the use of the Wong–Baker FACES pain rating scale as an appropriate self-reported pain assessment tool among children. It shows high sensitivity and validity, is simple to use, and is preferred by pediatric patients in comparison with other pain scales [31]. Existing data also support the use of the FLACC behavioral pain assessment scale across different populations and settings; further, it is reliable and sensitive to procedural pain in young children [32].

VR distraction was found to be effective in reducing pain and anxiety when compared to the standard methods of behavior management in the medical and dental fields. Gold et al. showed that VR significantly reduced acute procedural pain and anxiety during phlebotomy procedures [33]. Another study compared VR distraction to regular behavior management techniques and concluded that VR distraction reduced the pain unpleasantness of intrusive dental procedures including fillings and extractions [34]. In the present study, we compared the VR distraction to an active control (regular screen) and found that the use of VR distraction had no added benefit, neither on HR nor pain reduction, during infiltration anesthesia when compared with the classical audiovisual distraction using the regular screen. Those findings are in agreement with Al Halabi et al. who assessed the effect of VR distraction on pain and anxiety during inferior alveolar nerve blockage in pediatric patients [6]. On the other hand, Attar et al. compared the pain scores using video glasses to using an iPad and found that pain scores were higher in the video glasses group [23].

On the contrary, some studies found a positive impact of VR distraction on pain and anxiety [7, 13]. A study by Aminabadi et al. in 2012 showed a significant decrease in pain and anxiety scores when treating pediatric patients ages 4 to 6 years while being distracted using VR goggles [7]. Also, Panda in 2017 reported that the use of VR eyeglasses significantly reduced the pain felt by 6- to 8-year-old children during dental treatment [13]. In these studies, VR was used as a distraction method throughout the entire dental procedure, which included anesthesia and restorative or pulpal therapy. The difference in results could be explained by the longer duration of VR distraction, which allows for a more immersive effect on subjects. Another justification of lowered experienced pain could be that dental treatment was provided after successful anesthesia was established, making the remaining duration of VR distraction nonpainful. In addition, the age group in these studies was younger than in our study sample.

To the best of our knowledge, few studies have assessed the effects of age and gender on pain and anxiety when using VR distraction. Kaur et al. evaluated the effects of audiovisual distraction on anxiety among 4- to 6- vs. 6- to 8-year-old children and found that it was effective in alleviating anxiety in both age groups [25]. In our study, females were found to have a higher FLACC behavioral pain assessment scale scores compared with males regardless of the type of distraction used. While gender did not have an effect on the Wong–Baker FACES pain rating scale, but since it is a self-reported scale, there is a chance that subjects, especially the older group, downplayed their pain assessment to look better.

The strengths of our study include randomization, which aimed at controlling for known and unknown confounding factors. The study was designed to ensure allocation concealment to eliminate possible selection bias. Also, the calibration of examiners before the start of the study can be considered a strength. Furthermore, the study’s statistical analysis considered the effects of interaction and confounding. However, our study had limitations. The FLACC behavioral pain assessment scale requires direct observation; therefore, there was a lack of blinding of the investigators who assessed pain. Also, the buccal infiltration anesthesia was administered by different clinicians, but all were experienced (postgraduate pediatrics dental students and interns), which may have increased variability between subjects. However, there was no significant difference in provider-type distribution between the test and control groups. Another limitation of the study is that assessment of VR distraction was performed during local anesthesia only. Therefore, further long-term randomized controlled clinical trials on a larger sample size should be conducted to assess VR distraction during other dental restorative, pulpal, and surgical procedures.

Future studies may consider using VR systems with integrated eye tracking and compare its effect in reducing pain and discomfort with the traditional VR systems in children during dental procedures. Advances in VR technology may have serious ramifications on the effectiveness of VR analgesia during dental procedures. Virtual reality gadgets with higher resolutions and wider field of views were found to be effective in significantly reducing thermal pain on the skin and the time spent thinking about the pain compared to low resolution and narrower field VR systems [35]. Additional potential areas of study in this field include the patient satisfaction and enjoyment with the virtual reality experience during dental visits.

## Conclusion

The utilization of virtual reality goggles has a similar effect to screen distraction on heart-rate levels and pain during buccal infiltration anesthesia among pediatric patients. Female subjects, younger subjects, and subjects with higher baseline heart rates were more likely to report higher pain scores during local anesthesia administration regardless of the type of distraction used. Further research is needed to assess the effect of virtual reality distraction during various dental procedures other than anesthesia.

## Data Availability

The dataset of the study can be made available by the corresponding author upon reasonable request.

## Abbreviations

AAPD: American Academy of Pediatric Dentistry
ANCOVA: Analysis of covariance
FLACC: The face, legs, activity, cry, consolability behavioral pain assessment scale
HR: Heart rate
KAUFD: King Abdulaziz University Faculty of dentistry
SD: Standard deviation
SE: Standard error
OR: Odds ratio
VR: Virtual reality
